# The importance of vitamin D in maternal and child health: a global perspective

**DOI:** 10.1186/s40985-017-0066-3

**Published:** 2017-09-01

**Authors:** M Fiscaletti, P Stewart, CF Munns

**Affiliations:** 1 Institute of Endocrinology, The Children’s Hospital at Westmead, Corner Hawkesbury Road and Hainsworth Street, Locked Bag 4001, Westmead, NSW 2145 Australia; 2 Discipline of Child and Adolescent Health, Sydney Medical School, University of Sydney, Sydney, Australia

**Keywords:** Vitamin D, 25-Hydroxycholecalciferol, Vitamin D deficiency, Nutritional rickets, Review, Maternal health, Child health, Global public health

## Abstract

Vitamin D and calcium are important nutrients for skeletal growth and bone health. Children and pregnant women are particularly vulnerable to 25-hydroxy vitamin D deficiency (VDD). VDD, with or without dietary calcium deficiency, can lead to nutritional rickets (NR), osteomalacia, and disturbances in calcium homeostasis. Multiple studies have linked VDD to adverse health outcomes in both children and pregnant women that extend beyond bone health. VDD remains an important global public health concern, and an important differentiation must be made between the impact of VDD on children and adults. Reports of increased incidence of NR continue to emerge. NR is an entirely preventable condition, which could be eradicated in infants and children worldwide with adequate vitamin D and calcium supplementation. The desire and necessity to put in place systems for preventing this potentially devastating pediatric disease should not elicit dispute. VDD and NR are global public health issues that require a collaborative, multi-level approach for the implementation of feasible preventative strategies. This review highlights the history, risk factors, and controversies related to VDD during pregnancy and childhood with a particular focus on global NR prevention.

## Background

Vitamin D and calcium are essential for adequate health throughout the lifespan. Pregnant women and children however are particularly vulnerable to vitamin D deficiency. Nutritional rickets is a devastating neuromuscular disease due to vitamin D status and/or calcium deficiency and continues to be an important global health problem. Public health strategies such as food fortification and supplementation are not universal, and their implementation has proven difficult despite the increasing evidence of the role of vitamin D status on health and disease status.

## Introduction

Vitamin D status and calcium are important nutrients for skeletal growth and bone health. Children and pregnant women are particularly vulnerable to vitamin D deficiency (VDD). Multiple studies have linked VDD to adverse health outcomes in both children and pregnant women. Despite the abundance of scientific studies in the last decades regarding the possible extra-skeletal role of vitamin D status, the evidence remains mostly inconsistent. What is not controversial, however, is that VDD, with or without dietary calcium deficiency, can lead to nutritional rickets (NR), disturbances in calcium homeostasis, and osteomalacia.

**Fig. 1:**
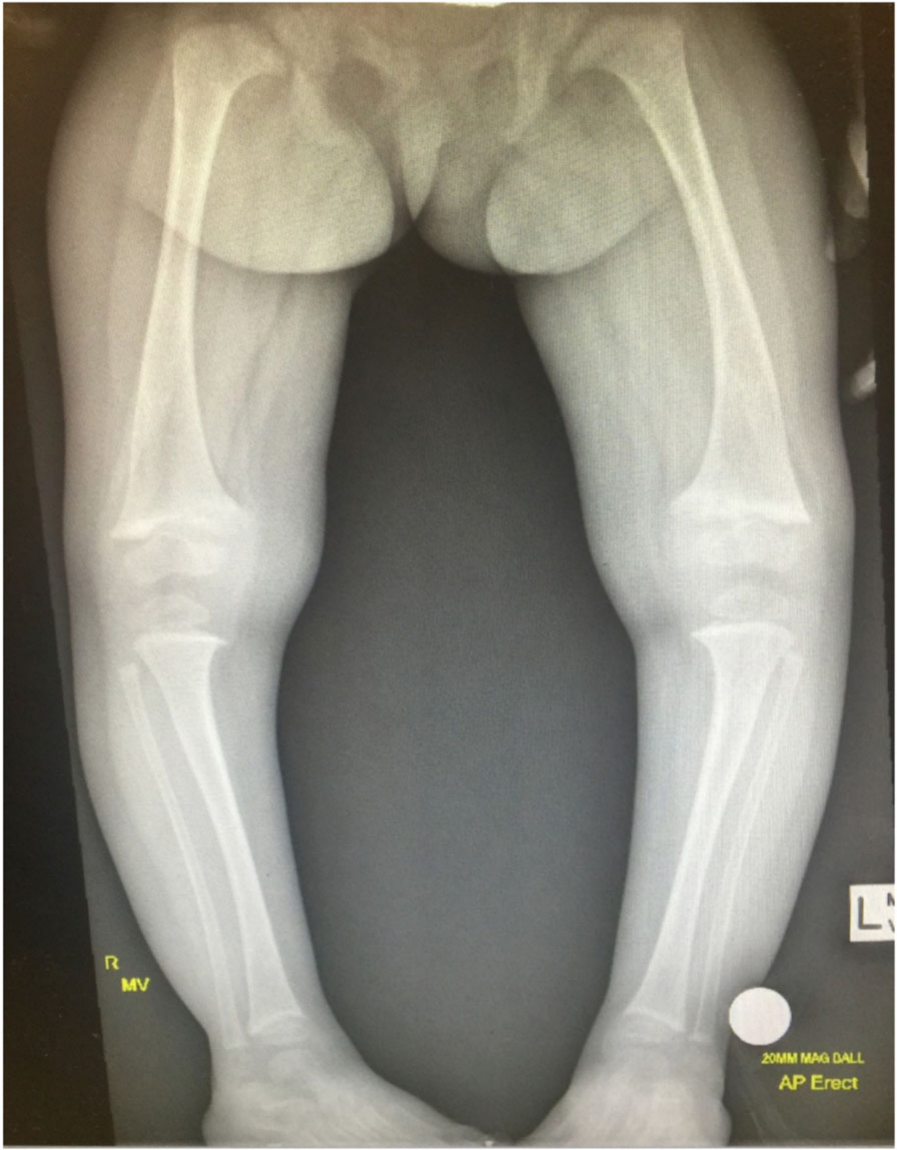
X-ray of the lower extremities of a child with nutritional rickets. Classic radiological signs of rickets are seen including cupping, fraying, and widening of the growth plates and bowing of the diaphyses

NR is a pediatric condition where chondrocyte differentiation and bone mineralization at the growth plates are defective and can lead to short stature and skeletal deformities [1, 2]. Skeletal features of NR can be seen in Fig. 1. Osteomalacia is defective mineralization of the osteoid in cortical and trabecular bone [2]. Although this is a term often used to describe the demineralization caused by VDD in adults, it is important to note that this also describes histological changes that can be seen in children with NR [2]. For simplicity, NR will refer to both the histological changes and demineralization at all segments of the bone and will exclude heritable disorders of vitamin D metabolism and congenital or acquired hypophosphatemic rickets.

Consequences of NR extend beyond bone. Although uncommon, NR-related hypocalcemia can lead to seizures, tetany, generalized weakness, cardiomyopathy, and raised intracranial pressure, all of which can have devastating consequences. While the global prevalence/incidence of these non-skeletal manifestations remains unknown, there have been numerous published case reports describing cardiomyopathy associated with rickets [3–8] and rachitic hypocalcemic seizures [9, 10]. In a retrospective case series aimed at reviewing the prevalence of VDD associated with pediatric cardiomyopathy in South East England, 16 infants were identified in a 6-year chart review [3]. All infants were from a dark-skinned ethnic minority group, exclusively breastfed and presented with a median 25-hydroxy vitamin D (25OHD) status of < 20 nmol/L (VDD was defined as < 35 nmol/L) and radiological signs of rickets [3]. In another study capturing the incidence of VDD rickets in Canada, the majority of pediatric cases showed clinically important morbidity at diagnosis and almost 20% of cases presented with hypocalcemic seizures [11].

Multiple guidelines and consensus statements on NR [1, 12] and VDD [13–17] have been published in the last decade recommending supplementation and fortification. Unfortunately, implementation of public health policy changes and interventions have been limited [18], and NR eradication remains elusive. Long-standing debates about exact definitions of vitamin D status coupled with recent high-profile publications arguing against global VDD pandemic [19–21] have led some to question the importance of vitamin D supplementation. It is unfortunate that this debate, which has been led primarily by adult physicians, has failed to differentiate the consequences of VDD on children from adults. What should remain clear and uncontroversial are the devastating pediatric consequences associated with VDD that extend beyond bone such as cardiomyopathy and seizures.

This manuscript will review the history, risk factors, and controversies related to VDD during pregnancy and childhood. Additionally, this paper will focus on NR prevention and management as they pertain to global maternal and child health and examine issues such as fortification, supplementation, and individualized care for vulnerable groups.

### Historical epidemiological fluctuations of VDD

With the start of the industrial revolution, urbanization, over-crowding, and unsanitary conditions contributed to an unprecedented rise in NR [2, 22, 23]. Chronic bony deformities resulted in long-term morbidity into adulthood and triggered an increase in the rates of cesarean sections in order to safely deliver children born to women with improper bone growth and rachitic pelvises [24]. Once pathophysiology and treatment of NR was described, government led public awareness and recommendations for vitamin D supplementation and fortification were implemented [25]. During the 1930s and 1940s, nutrient fortification of staple foods, including vitamin D fortification, in the USA, Canada, and UK were applied [26]. NR incidence decreased, only to rise again once those regulations waned in the post-war era [25, 26]. In Canada, it was not until the reintroduction of fortification, almost four decades later, that decline in NR resumed [23].

In developing countries, however, attempts to drop NR rates have proven difficult. Prevalence rates from Africa, Middle East, and Asia far exceed those in Western countries and are a major problem in infants [27, 28] and children [29]. Dietary sources of calcium are variable across different regions and are habitually low in developing countries where dairy products are scarce [1]. Calcium deficiency alone (i.e., with normal 25-hydroxy vitamin D levels) can still predispose a child to NR. This is evident from cases in developing countries where vitamin D sufficient children still present with NR [1, 30, 31].

Although NR remains a relatively rare disease, there are recent reports of increased incidence in the USA [32, 33], Canada [11], Denmark [34], Australia [35], and UK [36]. In the UK, rates for NR are at five-decade high [36] and clinically severe cases of VDD are still occurring [37]. Cases are not limited to countries situated at high latitudes with little sunlight. Even in developed, sub-tropic countries like Australia, increased cases of hypocalcemic seizures and musculoskeletal deformities from VDD have been reported [38]. Despite much progress over the last century, many risk factors for symptomatic VDD remain.

### Prevalence of vitamin D insufficiency and deficiency

A recent cohort study of vitamin D status in pregnant white-skinned women and their infants in North West England revealed that 27% of mothers had insufficient (< 50 nmol/L) and 7% had deficient (< 25 nmol/L) 25OHD levels during pregnancy; their levels dropped in 48 and 11% of cases, respectively, 4 months after delivery [39]. In this same study, 24% of infants had 25OHD levels between 25 and 50 nmol/L and 13% had 25OHD levels < 25 nmol/L at 4 months of age [39]. Another prospective study from the UK looking at micronutrient status in pregnant teenagers found that 30% of the participants had 25OHD levels below 25 nmol/L [40].

### Determinant of plasma 25OHD concentrations

The major circulating form of vitamin D (25OHD) is synthesized in the skin as cholecalciferol (vitamin D3) with very few food sources containing either ergocalciferol (vitamin D2) or cholecalciferol [41]. Endogenous skin synthesis requires the skin be exposed to ultraviolet B (UVB) light (290–315 nm wavelengths). Apart from fortified foods, dietary intake of vitamin D is limited. Table 1 summarizes the many factors that influence both environmental and dietary contributions of vitamin D in humans.

**Table 1:** Risk factors for low 25OHD concentrations

Risk factors that limit skin exposure to UVB rays	
Latitudes above 40° north	
Winter season	
Exposure in early morning and evening (before 10 AM, after 4 PM)	
Cloud cover and atmospheric pollution	
Limited time spent outdoors	
Customary dress that conceals large portions of the body	
Sunscreen use	
Dark skin pigmentation	
Older age	
Risk factors that limit dietary exposure to vitamin D	
Low dietary intake of oily fish and egg yolks	
Vegetarian diets	
Low/no dietary intake of vitamin D fortified foods	
Exclusive breastfeeding in infants	
No intake of vitamin D supplements	
Other risk factors that alter vitamin D supply or metabolism	
Vitamin D status of infant depends on vitamin D status of mother during pregnancy	
Low dietary calcium intake	
Obesity	
Genetic factors that affect vitamin D physiology and requirements	
Poor renal function	
Liver disease and cholestasis	
Chronic disease	
Malabsorption (coeliac, inflammatory bowel disease, cystic fibrosis, etc.)	

Vitamin D status is also influenced by non-modifiable genetic factors implicated in vitamin D metabolism. These can include inter-individual differences in vitamin D/calcium absorption and transport, or genetic polymorphisms of proteins and receptors involved with vitamin D. Certain polymorphisms of the vitamin D receptor, for example, have been found to lead to inter-individual differences in bone mineral density [42].

### VDD during pregnancy

**Fig. 2:**
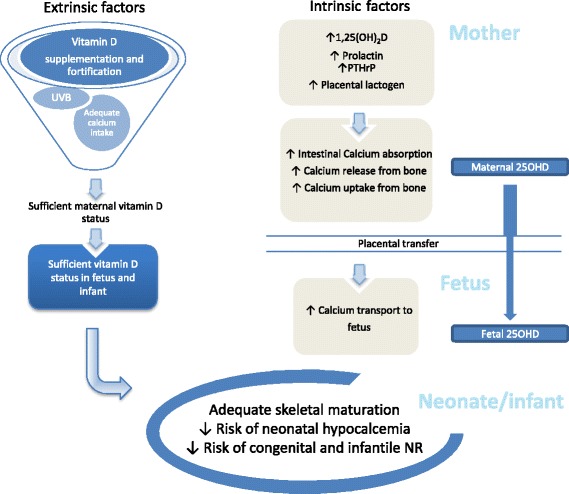
Schematic representation of some maternal factors and physiological changes during pregnancy that optimize bone health in offspring. Increased calcium transport to fetus and sufficient maternal vitamin D status result in adequate skeletal maturation, decreased risk of neonatal hypocalcemia, and decreased risk of congenital and infantile NR. Maternal 25OHD likely crosses the placenta resulting in fetal levels that approximate maternal levels. 1,25(OH)_2_D, on the contrary, is not thought to cross the placenta. 1,25(OH)_2_D 1,25-dihydroxycholecalciferol, UVB ultraviolet B, NR nutritional rickets

Multiple physiological adaptations occur during pregnancy to ensure rapid growth and mineralization of the fetal skeleton. The full review of maternal physiological mechanisms that occur during pregnancy to optimize fetal skeletal development is beyond the scope of this paper. A schematic summary is provided in Fig. 2. In order for the mother to provide the 30 g of calcium required for adequate fetal bone development, maternal intestinal calcium absorption and calcium resorption from bones are increased [43]. A small loss in bone mineral content may occur during pregnancy; however, retrospective studies have not shown that parity is a risk factor for osteoporosis in women with normal bone turnover [44]. In pregnant women, gravidity does not exacerbate pre-existing osteomalacia or VDD [45]. Maternal vitamin D status is, however, associated with infant vitamin D status [46, 47].

Pre-pregnancy or peri-pregnancy VDD in the mother can have important ramifications for the fetus and infant. Reports of high prevalence of VDD below 25 nmol/L in pregnant women from ethnic minority groups living in temperate climates ranges 60–80% [45]. Maternal vitamin D status reflects fetal and neonatal status. Significant linear correlations have been shown between maternal and umbilical cord plasma levels of 25OHD with cord levels being lower than maternal ones and pointing to a placental diffusion barrier or differences in binding protein affinities [46–48]. Consequently, maternal VDD can be transmitted to the fetus and newborn and, depending on severity, can lead to both acute and delayed consequences.

### VDD during pregnancy and maternal outcomes

Vitamin D status during pregnancy and its impact on maternal outcomes have been extensively studied. While observational studies have suggested that VDD during pregnancy is associated with increased risk of adverse maternal outcomes such as pre-eclampsia, gestational diabetes mellitus (GDM), and cesarean section, interventional studies have reported conflicting results [49].

#### Pre-eclampsia

Pre-eclampsia, a condition defined by new-onset gestational hypertension and proteinuria, after the 20th week of gestation, is more likely to occur in women with VDD [50–52]. RCTs examining vitamin D supplementation and increased pre-eclampsia outcome have reported conflicting results in the past. Two recent systematic reviews, including a Cochrane review, critically appraising data from five RCTs involving almost 1300 women, have judged that women who receive daily vitamin D supplements with and without calcium supplements have higher 25OHD levels and lower risk of pre-eclampsia compared to women receiving no intervention or placebo [49, 53]. Low-quality evidence from two RCTs looking at vitamin D supplementation alone during pregnancy compared to placebo or no intervention found a pre-eclampsia risk ratio (RR) of 0.52 (95% CI 0.25–1.05) [53]. It is important to note that supplementation in these two RCTs was heterogeneous, specifically daily 400 IU of cholecalciferol in Asemi et al.’s trial [54] compared to a single dose of 60,000 IU, two doses of 120,000 IU, or four doses of 120,000 in the treatment arm of the Sablok et al.’s study [55]. The RR of pre-eclampsia was even lower when calcium was also supplemented. Moderate-quality evidence from data of three RCTs showed that women who received vitamin D and calcium supplementation also had an even lower risk of pre-eclampsia compared to those without intervention (RR 0.51; 95% CI 0.32–0.80) [53].

#### Gestational diabetes mellitus (GDM)

There are inconsistent data regarding maternal VDD and increased risk for GDM [50]. In two cross-sectional studies, severe VDD, as defined by levels < 12.5 nmol/L, was significantly higher in women with GDM [51, 56]. As is the case with all observational studies, causation cannot be implied, since multiple confounding factors both measured and unmeasured, such as ethnicity, genetics, physiological variability, and adiposity can confound the relationship. An RCT examining the effects of vitamin D supplementation on maternal glucose metabolism during pregnancy found that starting high-dose vitamin D (5000 IU daily) during the second trimester did not normalize glucose levels on oral glucose tolerance test but was highly effective at preventing neonatal VDD compared to low-dose (400 IU daily) vitamin D supplementation [57].

#### Other outcomes

Various other maternal outcomes and vitamin D status during pregnancy have been assessed. Reduced rates of cesarean section have been inconsistently associated with higher 25OHD levels in recent Spanish and Asian observational studies [58–60].

Other adverse maternal outcomes associated have been linked to VDD. One study found women seeking medical help for infertility had deficient 25OHD levels [61]. Other associations between low prenatal and perinatal maternal vitamin D concentrations and multiple sclerosis, cancer, insulin-dependent diabetes mellitus, and schizophrenia have also been reported [62]. Recent investigations studying the link between 25OHD levels and recurrent pregnancy loss and post-partum depression have also been recently studied without any clear evidence to support a causal relationship [49].

### VDD during pregnancy and infant/child outcomes

Pregnant women should receive 600 IU of daily supplemental vitamin D to ensure sufficient maternal 25OHD levels and to prevent complications in the infant [1, 63]. VDD during pregnancy and multiple infant-related outcomes have been recurrently studied.

#### Congenital rickets and hypocalcemic complications

Craniotabes and congenital rickets, defined as the presence of rickets in the first month of life [1], are linked to VDD during pregnancy [47]. Other non-osseous signs and symptoms in infants can be particularly severe and troubling and include neurological complications such as hypocalcemic seizures, increased intracranial pressure, muscle weakness, and tetany. Evidence from interventional and observational studies report an association between low maternal vitamin D status and abnormal infant outcomes such as elevated blood alkaline phosphatase [64], larger fontanelle size at birth [65], and neonatal hypocalcemia [65–68]. Moreover, some hypocalcemia complications can be life-threatening including dilated cardiomyopathy leading to heart failure, arrhythmia, and cardiac arrest.

#### Anthropometry

There is insufficient and conflicting evidence that maternal vitamin D supplementation during pregnancy can alter birth anthropometry [1]. Well-conducted RCTs have shown that vitamin D supplementation during pregnancy ranging from daily doses of 800 to 4000 IU or single dose regimens of 100,000 or 200,000 IU of cholecalciferol commencing in the second or third trimester did not lead to anthropometric changes [65, 69–72].

Conversely, moderate-quality evidence from three trials examining the role of vitamin D supplementation during pregnancy in almost 500 women revealed decreased frequency of low birth weight (< 2500 g) in infants from supplemented mothers compared to those who had no intervention or placebo during pregnancy (RR 0.4, 95% CI 0.24–0.67) [53]. Furthermore, an RCT looking at vitamin D supplementation (35,000 IU/week) in mothers during their third trimester of pregnancy revealed enhanced early postnatal linear growth in Bangladeshi infants from supplemented mothers compared to infants from mothers who had received placebo [73]. Further data are needed to explore the true relationship between maternal vitamin D supplementation and infant anthropometry. These differing results in infant anthropometry may suggest that supplementation during pregnancy of vitamin D deficient women may be more important in underdeveloped nations.

#### Prematurity

Similarly, three recent RCTs of moderate quality have reported that vitamin D supplementation during pregnancy decreases the risk of preterm birth in supplemented women compared to those receiving placebo or no intervention [54, 55, 74] and this was confirmed in a meta-analysis with an average RR of 0.36 (95% CI 0.14–0.93) [53]. Conversely, combined results from three RCTs examining both vitamin D and calcium supplementation in pregnant women revealed increased rates of preterm birth in supplemented women (RR 1.57; 95% CI 1.02–2.43) [53]. The reasons for this remain unclear. Guarded interpretation of these results is emphasized due to the small number and limited quality of these trials.

#### Infant bone mass

Fetal and infant bone mass may be influenced by maternal VDD during pregnancy [75]. However, the evidence from multiple observational and interventional studies was recently reviewed by an international panel of bone experts and found to be inconclusive [1].

#### Other infant/child outcomes

Multiple studies examining the relationship between VDD during pregnancy and non-skeletal infant/child outcomes have been reported. Observational studies have suggested possible links with respiratory infections, immunity, and autism [49]; however, well-conducted, large interventional trials are lacking. One RCT studying the effect of maternal high-dose vitamin D supplementation during pregnancy on toddler wheezing or asthma diagnosis revealed a possible protective effect but failed to reach statistical significance [76].

### High-risk groups for VDD and NR

Preventing VDD in pregnant women remains a global imperative to prevent neonatal VDD, which can have severe and sometimes irreversible consequences [1, 77]. This is particularly important in high-risk groups.

#### Preterm infants

Prematurity increases the risk of VDD. Preterm birth amputates the time for adequate transplacental transfer of vitamin D and leads to deficient fetal vitamin D stores [78]. Globally, approximately 15 million children are born preterm each year [79], and survival rates for premature babies are at a historical high [80]. The majority of skeletal calcium and phosphorus deposition is accomplished in the third trimester of pregnancy; therefore, premature babies have low mineral stores. Additionally, they are born during a phase of rapid growth including rapid bone mineral accretion [43]. Difficulties in mineral accretion are compounded by poor early intake, frequent illness, prolonged immobility, and medications altering bone mineral homeostasis such as steroids and diuretics [43].

#### Term infants

Even term infants remain at risk for NR and VDD. Breast milk is the best choice for infants despite its low levels of vitamin D. One liter of breast milk contains a maximum of 25 IU vitamin D [81], well below the intake levels necessary to prevent NR. Mothers with additional risk factors, who exclusively breastfeed, are particularly at risk of having an infant with symptomatic VDD [28, 38, 82]. To prevent NR, breastfed infants should therefore be supplemented with 400 IU of vitamin D daily in their first year of life [1].

While infant formulas are often fortified with vitamin D, infants can still remain at risk for NR if they are born from 25OHD deficient mothers and/or consume less than 1 l of formula per day [11, 83]. Dark-skinned women living at higher latitudes are particularly at high risk for VDD [84, 85].

#### Immigrants and refugees

VDD during pregnancy occurs globally [86–89], particularly in migrant women from high-risk groups migrating to temperate climates [38, 90]. Globalization and recent social/political conflicts have caused a spike in migration across Europe and North America. Middle Eastern, African, and south-Asian migrants and their offspring that relocate to temperate climates are particularly at risk for VDD [91]. Increases in NR cases in sunny climates have mirrored immigration trends [38], where people with darker skin pigment and culture traditions that limit exposure to sunshine are at increased risk of VDD especially pregnant women and their children [92]. With the current refugee crisis, an elevated number of high-risk children will migrate to high-income countries with temperate climates and will be at even greater risk for vitamin D and/or calcium deficiency [1, 91, 92].

Geographical displacement can also trigger changes in diet, which could alter the course of developing NR. Knowing that there is an interaction between calcium intake and vitamin D status, changes in calcium intake may exacerbate or attenuate the NR. An asymptomatic child who previously had mildly insufficient vitamin D status and mildly insufficient calcium intake who then migrates to a country where his calcium intake is further reduced is likely to develop biochemical anomalies and/or NR.

#### Other groups at risk for VDD

Obesity and other chronic medical conditions can interfere with vitamin D absorption and metabolism. Sequestration of vitamin D in fat stores may explain an inverse association between obesity and 25OHD levels [93, 94]. With conditions that alter fat absorption are at risk for VDD and NR. This has been shown in children with Coeliac’s disease [95] but can apply to other malabsorption states (cystic fibrosis, inflammatory bowel disease, etc.) [93].

### Current controversies surrounding VDD

Debate over exact definition of VDD persists in the literature. Uniform distinctions between “deficient,” “insufficient,” and “sufficient” levels are important for accurate epidemiological and comparative data. However, published scientific debate over uniform definitions should not overshadow the importance of VDD in pregnant and pediatric populations. Studies whose only outcomes are 25OHD serum levels and their relationship to extra-skeletal benefits do not address the more pressing matters of bone complications in high-risk populations. What is not controversial is the importance of eradicating NR and other severe vitamin D deficient conditions in pediatric populations.

A recent high-profile publication argued that because of a misapplication of the Institute of Medicine (IOM) nutritional requirements, studies have inflated the prevalence of VDD [19]. The authors state that the Estimated Average Requirement (EAR), defined as the median of the distribution of population requirement (40 nmol/L), and not the Recommended Daily Allowance (RDA), should be used as the target intake to achieve vitamin D sufficiency in the population. By using RDA as the target for population-based 25OHD levels, defined as the nutritional requirements that meet 97.5% of the population’s needs and correspond to 25OHD levels beyond 50 nmol/L, the authors argue that many manuscripts have misclassified subjects as deficient and have inflated the prevalence of VDD [19]. While the author’s arguments may be valid when encompassing a general, healthy, adult population from high-income countries, some important issues need to be considered. First, vitamin D requirements are unlikely to be stable throughout the lifespan. Second, seasonal fluctuations of 25OHD levels are not negligible. Third, there is inherent variability in vitamin D intake and metabolism. Finally, in certain populations, concomitant calcium intake may be decreased. Aiming for 25OHD levels as per the EAR in populations with multiple risk factors for VDD might be imprudent. In children, PTH was shown to increase when 25OHD levels drop below 34 nmol/L [96] and seasonal fluctuations have been shown to decrease 25OHD levels to a nadir between 13 and 24 nmol/L [92]. In a large adult American cohort, seasonal variations in intact PTH levels appear to follow a slightly delayed but inverted pattern relative to 25OHD levels and VDD with secondary hyperparathyroidism was found in a substantial proportion of the population [97]. High serum PTH levels will lead to phosphaturia, and low serum phosphate levels and if sustained will compromise bone mineralization and lead to osteomalacia and rickets. An explanation of the historical relationship between diagnosing 25OHD deficiency and PTH levels has been recently published [98]. After extensive review of the literature, the global consensus on NR strongly recommended that levels between 30 and 50 nmol/L were insufficient and that in order to prevent NR, it is important to maintain 25OHD levels beyond 50 nmol/L to counteract the plunge seen with seasonal variations [1]. Nevertheless, defining a clinically significant 25OHD level is challenging due to inter-individual variability in vitamin D metabolism.

The real controversies that need to be addressed are the benefits of 25OHD levels above 50 nmol/L during childhood for the benefits of skeletal health. With adequate calcium intake, NR is unlikely to occur if 25OHD levels are beyond 34 nmol/L [99]. It is not controversial to want to prevent NR and hypocalcemic complications. The recent global consensus guidelines on NR supported the IOM definitions and Pediatric Endocrine Society [16] of vitamin D thresholds, specifically < 30 nmol/L as deficient, 30–50 nmol/L insufficient, and > 50 nmol/L sufficient. These thresholds were selected based upon associations between 25OHD levels and clinical and biochemical evidence of rickets [1].

### Recommendations

#### Pregnancy

The global consensus on NR recommended that all pregnant women should receive 600 IU/day of supplemental vitamin D to prevent both neonate and infant biochemical and radiographic signs of NR [1]. This is in keeping with recommendations from American College of Obstetrics and Gynecologists [100], National Institute for Health and Care Excellence [101], and Institute of Medicine [63] that also recommend supplementation in pregnancy.

#### Infants

There is international consensus regarding the vitamin D supplementation of breastfed infants. Owing to the low content of vitamin D in breast milk and the increased risk of NR among exclusively breastfed infants, multiple professional societies have recommended that breastfed infants require 400 IU/day of vitamin D supplementation [1, 16, 63, 102]. Formula-fed infants could still remain at risk of NR [11] despite government-mandated vitamin D fortification of infant formulas. Infant formulas provide 400 IU/l [93]. Infants who are born with low vitamin D status, have additional risk factors, or are not receiving 1 l of formula daily may still remain at risk for NR [11, 93]. The global consensus guidelines have strongly recommended that all infants, regardless of their mode of feeding, be supplemented with 400 IU/day from birth until 12 months of age based on high-quality evidence [1]. In prospective studies in Canada, China, and Turkey, there were no incident radiographically confirmed NR cases in children supplemented with 400 IU/day [11, 103, 104].

#### Children

Beyond the first year of life, vitamin D requirements increase to 600 IU/day. Multiple professional societies strongly recommend that all healthy children beyond 12 months achieve this nutritional intake either through diet or supplementation [1, 63, 105, 106]. Children with malabsorption or other chronic conditions that may alter vitamin D absorption/metabolism, children from high-risk groups, and those living in high latitudes may have vitamin D requirements beyond 600 IU/day [93, 106]. For the maximization of bone health in children, adequate calcium intake, weight-bearing exercise, and maintenance of healthy body weight are essential through puberty. [15]

### Policy and public health approaches are necessary, safe, and effective

In most western diets, few foods are an abundant source of vitamin D [23]. Accordingly, achieving recommended intake of vitamin D is unlikely to occur through diet alone, unless there is fortification of key food groups [23]. Vitamin D supplementation and fortification guidelines and their implementation vary widely around the world [107]. International recommendations for vitamin D intake during pregnancy and for infants and children are variable [108].

While many countries have public health policies regarding vitamin D supplementation, adherence to these programs is typically poor. A recent study assessing variations in infant and childhood vitamin D supplementation programs across Europe revealed that 96% of European countries had national policies for infant vitamin D supplementation; however, adherence was either moderate or low in almost half of the countries surveyed [109]. In the UK, awareness of and adherence to national recommendations for vitamin D supplementation remain problematic [95, 110, 111]. Similarities have been described in the USA [112, 113], where less than 15% of infants receiving both breast milk and infant formula were meeting their nutritional requirements of vitamin D. In Scandinavian countries, adherence is better but far from ideal, where 59 and 64% of breastfed infants receive supplements in Norway and Sweden respectively [114, 115]. A systematic review in adults examining the health effects and societal burden of vitamin D through epidemiological studies reveals that current fortification and supplementation policies are inadequate [18]. This lack of adherence is concerning, given the devastating bony outcomes for NR. Some authors have suggested supplementing lactating mothers with high doses of vitamin D such as 2000–6400 IU/day [116] or a single dose of 150,000 IU [117] to achieve desired 25OHD sufficiency in their infants. All regimens were effective in supplying vitamin D to infants, and daily doses up to 4000 IU were deemed likely safe by the authors [116, 117]. However, it was equally effective to supplement infants with 400 IU/d to achieve similar 25OHD levels as those whose mothers were supplemented with 2000 IU/day or higher vitamin D. Of note, no consensus guidelines or group has promoted using high doses of vitamin D supplementation in lactating women [1, 12, 53, 63].

Vitamin D supplementation during pregnancy, with doses ranging from 400 to 4000 IU/day, have been reported as safe. A multicentre, double blind, randomized, placebo-controlled trial reported that 1000 IU/day of vitamin D supplementation during pregnancy was effective in maintaining sufficient 25OHD levels safely [118]. Similar studies have corroborated the safety of vitamin D supplementation during pregnancy using doses at or above the IOM recommendations [57, 119, 120]. While judicious supplementation is both safe and effective, its implementation remains problematic and inconsistent in multiple regions. Fortification of staple foods can provide additional vitamin D intake in the general population.

As changing socio-political factors create an environment for likely increases in reported NR cases, a global strategy is imperative to eradicate this preventable condition. The global consensus guidelines confirmed that 25OHD and calcium deficiencies are common worldwide, lead to NR in pediatric populations, and are completely preventable with proper supplementation of all pregnant and lactating mothers and infants [1]. Although one study provided evidence for vitamin D supplementation in high-risk groups in the UK [121], more data examining cost-effective strategies of supplementation and food fortification programs, especially in high-risk groups, is necessary [1].

Evidence in favor of safe staple food fortification is available. Since the 1980s, Canadian law mandates vitamin D fortification of liquid milk products and margarine; consequently, Canadian NR rates have decreased significantly [23]. Implementation of governmental vitamin D recommendations for fortification coincides with declines in incidence of NR among ethnic children including Denmark [34], Canada [23], and the USA [22]. Interventional studies comparing milk and vitamin D fortified milk consumption have shown that vitamin D fortified milk can improve both vitamin D status and bone density in adolescent girls [122]. Despite increasing the general population’s vitamin D intake, a caveat of food fortification is its inability to specifically target groups most at risk for VDD if they do not consume adequate amounts of the fortified food in question [123, 124]. Biofortification of a variety of foods has been suggested as a means to increase vitamin D intake among all subgroups of the population [125]. Government policy to increase food fortification must be coupled with nutritional education via primary care providers and public awareness campaigns.

Supplementation should be implemented into primary health care, and fortification of staple foods should be done based on dietary patterns. Finally, programs that are supported by government funding are more likely to be widespread and effective. A public health approach is needed, safe, inexpensive, and effective. A study evaluating the effectiveness of a universal vitamin D supplementation program for pregnant and lactating women and young children in the inner city of Birmingham, UK, resulted in a 59% decrease in symptomatic VDD children under 5 years of age and a substantial increase in public awareness of VDD [126].

Importantly, fortification of food groups will not help infants who exclusively breast or formula feed. For this group, vitamin D supplementation of breastfed or formula fed children is required to ensure vitamin D status sufficiency and prevent NR.

An interpretive study of the scientific literature pertaining to optimizing 25OHD levels and extra-skeletal benefits concluded that increasing serum 25OHD levels is the most cost-effective way to reduce global mortality rates [127]. There are important gaps in population-based studies examining vitamin D status worldwide, particularly in South America, Africa, and South-East Asia [128]. Government funding should reflect this striking lack of data especially in pediatric groups who continue to experience important burden from the consequences of VDD.

## Conclusion

The last decade has seen vigorous scientific debates and controversies apropos VDD, its diagnosis, significance, and supplementation for non-skeletal disease in adults, pregnant women, and children. Consistent evidence from RCTs comparing different vitamin D supplementation to placebo or no intervention has failed to show statistically significant differences in maternal or fetal outcomes [119, 129]. This likely reflects the association between poor health and concurrent low vitamin D status rather than a causal relationship.

As the scientific community continues to search for clearer data on these important issues, we must not neglect to emphasize the importance of NR eradication. The most undisputed consequences of VDD are also the most severe, and they occur in children. VDD causes NR. NR is completely preventable. A collaborative, international, multi-level approach is not only ideal and essential but has been proven feasible in preventing this potentially devastating pediatric disease.

## Abbreviations

1,25(OH)_2_D: 1,25-Dihydroxycholecalciferol
25OHD: 25-Hydroxy vitamin D
EAR: Estimated Average Requirement
NR: Nutritional rickets
RCT: Randomized controlled trial
RDA: Recommended Daily Allowance
RR: Risk ratio
UVB: Ultraviolet B radiation
VDD: Vitamin D deficiency

## References

[1] Munns CF, Shaw N, Kiely M, Specker BL, Thacher TD, Ozono K, Michigami T, Tiosano D, Mughal MZ, Makitie O (2016). Global consensus recommendations on prevention and management of nutritional rickets. J Clin Endocrinol Metab.

[2] Pettifor JM, Glorieux FH, Pettifor JM, H J (2012). Nutritonal rickets. Pediatric bone: biology and diseases.

[3] Maiya S, Sullivan I, Allgrove J, Yates R, Malone M, Brain C, Archer N, Mok Q, Daubeney P, Tulloh R (2008). Hypocalcaemia and vitamin D deficiency: an important, but preventable, cause of life-threatening infant heart failure. Heart.

[4] Elidrissy AT, Alharbi KM, Mufid M, Almezeni I: Rachitic hypocalcemic cardiomyopathy in an infant. J Saudi Heart Assoc 2017, 29(2):143-147. doi: 110.1016/j.jsha.2016.1005.1001. Epub 2016 Jun 1011. 10.1016/j.jsha.2016.05.001 PMC5366663 28373790

[5] Brown J, Nunez S, Russell M, Spurney C (2009). Hypocalcemic rickets and dilated cardiomyopathy: case reports and review of literature. Pediatr Cardiol.

[6] Price DI, Stanford LC, Braden DS, Ebeid MR, Smith JC (2003). Hypocalcemic rickets: an unusual cause of dilated cardiomyopathy. Pediatr Cardiol.

[7] Yilmaz O, Olgun H, Ciftel M, Kilic O, Kartal I, Iskenderoglu NY, Laloglu F, Ceviz N: Dilated cardiomyopathy secondary to rickets-related hypocalcaemia: eight case reports and a review of the literature. Cardiol Young 2015, 25(2):261-266. doi: 210.1017/S1047951113002023. Epub 1047951113002013 Dec 1047951113002017. 10.1017/S1047951113002023 24345686

[8] Verma S, Khadwal A, Chopra K, Rohit M, Singhi S: Hypocalcemia nutritional rickets: a curable cause of dilated cardiomyopathy. J Trop Pediatr 2011, 57(2):126-128. doi: 110.1093/tropej/fmq1044. Epub 2010 Jun 1015. 10.1093/tropej/fmq044 20554514

[9] Orbak Z, Karacan M, Doneray H, Karakelleoglu C (2007). Congenital rickets presenting with hypocalcaemic seizures. West Indian Med J.

[10] Ahmed I, Atiq M, Iqbal J, Khurshid M, Whittaker P (1995). Vitamin D deficiency rickets in breast-fed infants presenting with hypocalcaemic seizures. Acta Paediatr.

[11] Ward LM, Gaboury I, Ladhani M, Zlotkin S (2007). Vitamin D-deficiency rickets among children in Canada. CMAJ.

[12] Wagner CL, Greer FR. Section on Breastfeeding and Committee on Nutrition. Prevention of rickets and vitamin D deficiency in infants, children, and adolescents. Pediatrics. 2008;122(5):1142–52. 10.1542/peds.2008-1862 18977996

[13] Braegger C, Campoy C, Colomb V, Decsi T (2013). Vitamin D in the healthy European paediatric population. JPGN.

[14] WHO (2012). Guideline: vitamin D supplementation in pregnant women.

[15] Golden NH, Abrams SA. Committee on Nutrition. Optimizing bone health in children and adolescents. Pediatrics. 2014;134(4):e1229–43. 10.1542/peds.2014-2173 25266429

[16] Misra M, Pacaud D, Petryk A (2008). Vitamin D deficiency in children and its management: review of current knowledge and recommendations. Pediatrics.

[17] Prentice A: Vitamin D and health. In. Edited by Nutrition SACo. England: Public Health England; 2016.

[18] Ben-Shoshan M (2012). Vitamin D deficiency/insufficiency and challenges in developing global vitamin D fortification and supplementation policy in adults. Int J Vitam Nutr Res.

[19] Manson JE, Brannon PM, Rosen CJ, Taylor CL (2016). Vitamin D deficiency—is there really a pandemic?. N Engl J Med.

[20] Shah D, Gupta P (2015). Vitamin D deficiency: is the pandemic for real?. Indian J Community Med.

[21] Ferrari R, Prosser C (2016). Testing vitamin D levels and choosing wisely. JAMA Intern Med.

[22] Rajakumar K (2003). Vitamin D, cod-liver oil, sunlight, and rickets: a historical perspective. Pediatrics.

[23] Holick MF (2006). Resurrection of vitamin D deficiency and rickets. J Clin Invest.

[24] Cesarean Section - A Brief History. https://www.nlm.nih.gov/exhibition/cesarean/part3.html.

[25] Holick MF (2010). The vitamin D deficiency pandemic: a forgotten hormone important for health. Public Health Rev.

[26] Tulchinsky TH. Micronutrient deficiency conditions: global health issues. Public Health Reviews. 2010;32(1):243–55. 10.1186/s40985-017-0071-6 PMC5809998 29451564

[27] Kazemi A, Sharifi F, Jafari N, Mousavinasab N (2009). High prevalence of vitamin D deficiency among pregnant women and their newborns in an Iranian population. J Women’s Health (Larchmt).

[28] Dawodu A, Agarwal M, Sankarankutty M, Hardy D, Kochiyil J, Badrinath P (2005). Higher prevalence of vitamin D deficiency in mothers of rachitic than nonrachitic children. J Pediatr.

[29] Rabbani A, Alavian SM, Motlagh ME (2009). Vitamin D insufficiency among children and adolescents living in Tehran. Iran J Trop Pediatr.

[30] Thacher TD, Fischer PR, Pettifor JM, Lawson JO, Isichei CO, Reading JC, Chan GM (1999). A comparison of calcium, vitamin D, or both for nutritional rickets in Nigerian children. N Engl J Med.

[31] Oginni LM, Worsfold M, Oyelami OA, Sharp CA, Powell DE, Davie MW (1996). Etiology of rickets in Nigerian children. J Pediatr.

[32] Kreiter SR, Schwartz RP, Kirkman HN, Charlton PA, Calikoglu AS, Davenport ML (2000). Nutritional rickets in African American breast-fed infants. J Pediatr.

[33] Thacher TD, Fischer PR, Tebben PJ, Singh RJ, Cha SS, Maxson JA, Yawn BP (2013). Increasing incidence of nutritional rickets: a population-based study in Olmsted County, Minnesota. Mayo Clin Proc.

[34] Beck-Nielsen SS, Brock-Jacobsen B, Gram J, Brixen K, Jensen TK (2009). Incidence and prevalence of nutritional and hereditary rickets in southern Denmark. Eur J Endocrinol.

[35] Munns CF, Simm PJ, Rodda CP, Garnett SP, Zacharin MR, Ward LM, Geddes J, Cherian S, Zurynski Y, Cowell CT (2012). Incidence of vitamin D deficiency rickets among Australian children: an Australian paediatric surveillance unit study. Med J Aust.

[36] Goldacre M, Hall N, Yeates DG (2014). Hospitalisation for children with rickets in England: a historical perspective. Lancet.

[37] Shaw NJ, Pal BR (2002). Vitamin D deficiency in UK Asian families: activating a new concern. Arch Dis Child.

[38] Robinson PD, Hogler W, Craig ME, Verge CF, Walker JL, Piper AC, Woodhead HJ, Cowell CT, Ambler GR (2006). The re-emerging burden of rickets: a decade of experience from Sydney. Arch Dis Child.

[39] Emmerson AJB, Dockery K, Mughal MZ, Roberts SA, Tower CL, Berry JL (2017). Vitamin D status of white pregnant women and infants at birth and 4 months in north West England: a cohort study. Matern Child Nutr.

[40] Baker PN, Wheeler SJ, Sanders TA, Thomas JE, Hutchinson CJ, Clarke K, Berry JL, Jones RL, Seed PT, Poston L: A prospective study of micronutrient status in adolescent pregnancy. Am J Clin Nutr 2009, 89(4):1114-1124. doi: 1110.3945/ajcn.2008.27097. Epub 22009 Feb 27025. 10.3945/ajcn.2008.27097 19244368

[41] Molina PE (2013). Parathyroid gland and calcium and phosphate regulation. Endocrine physiology.

[42] Casado-Diaz A, Cuenca-Acevedo R, Navarro-Valverde C, Diaz-Molina C, Caballero-Villarraso J, Santiago-Mora R, Dorado G, Quesada-Gomez JM (2013). Vitamin D status and the Cdx-2 polymorphism of the vitamin D receptor gene are determining factors of bone mineral density in young healthy postmenopausal women. J Steroid Biochem Mol Biol.

[43] Bishop N, Fewtrell M, Harvey N, Glorieux FH, Pettifor JM, H J (2012). Metabolic bone disease in the neonatal period and its later sequelae. Pediatric bone: biology and diseases.

[44] Kovacs CS (2011). Calcium and bone metabolism disorders during pregnancy and lactation. Endocrinol Metab Clin N Am.

[45] Prentice A (2008). Vitamin D deficiency: a global perspective. Nutr Rev.

[46] Kovacs CS (2008). Vitamin D in pregnancy and lactation: maternal, fetal, and neonatal outcomes from human and animal studies. Am J Clin Nutr.

[47] Prentice A, Glorieux FH, Pettifor JM (2012). Pregnancy and lactation. Pediatric bone: biology and diseases.

[48] Wieland P, Fischer JA, Trechsel U, Roth HR, Vetter K, Schneider H, Huch A (1980). Perinatal parathyroid hormone, vitamin D metabolites, and calcitonin in man. Am J Phys.

[49] Agarwal S, Kovilam O, Agrawal DK (2016). Vitamin D and its impact on maternal-fetal outcomes in pregnancy: a critical review. Crit Rev Food Sci Nutr.

[50] Harvey NC, Holroyd C, Ntani G, Javaid K, Cooper P, Moon R, Cole Z, Tinati T, Godfrey K, Dennison E (2014). Vitamin D supplementation in pregnancy: a systematic review. Health Technol Assess.

[51] Bener A, Al-Hamaq AO, Saleh NM (2013). Association between vitamin D insufficiency and adverse pregnancy outcome: global comparisons. Int J Womens Health.

[52] Achkar M, Dodds L, Giguère Y, Forest JC, Armson BA, Woolcott C, Agellon S, Spencer A, Weiler HA (2015). Vitamin D status in early pregnancy and risk of preeclampsia. Am J Obstet Gynecol.

[53] De-Regil LM, Palacios C, Lombardo LK, Peña-Rosas JP: Vitamin D supplementation for women during pregnancy. Cochrane Database Syst Rev. 2016;14(1):CD008873. doi:10.1002/14651858.CD008873.pub3. 10.1002/14651858.CD008873.pub3 26765344

[54] Asemi Z, Samimi M, Tabassi Z, Shakeri H, Esmaillzadeh A (2013). Vitamin D supplementation affects serum high-sensitivity C-reactive protein, insulin resistance, and biomarkers of oxidative stress in pregnant women. J Nutr.

[55] Sablok A, Batra A, Thariani K (2015). Supplementation of vitamin D in pregnancy and its correlation with feto-maternal outcome. Clin Endocrinol.

[56] Maghbooli Z, Hossein-Nezhad A, Karimi F (2008). Correlation between vitamin D3 deficiency and insulin resistance in pregnancy. Diabetes Metab Res Rev.

[57] Yap C, Cheung NW, Gunton JE, Athayde N, Munns CF, Duke A, McLean M (2014). Vitamin D supplementation and the effects on glucose metabolism during pregnancy: a randomized controlled trial. Diabetes Care.

[58] Rodriguez A, García-Esteban R, Basterretxea M (2015). Associations of maternal circulating 25-hydroxyvitamin D3 concentration with pregnancy and birth outcomes. BJOG.

[59] Sebastian A, Vijayaselvi R, Nandeibam Y, Natarajan M (2015). A case control study to evaluate the association between primary cesarean section for dystocia and vitamin D deficiency. J Clin Diagn Res.

[60] Gernand AD, Klebanoff MA, Simhan HN, Bodnar LM (2015). Maternal vitamin D status, prolonged labor, cesarean delivery and instrumental delivery in an era with a low cesarean rate. J Perinatol.

[61] Pagliardini L, Vigano P, Molgora M, Persico P, Salonia A, Vailati SH, Paffoni A, Somigliana E, Papaleo E, Candiani M (2015). High prevalence of vitamin D deficiency in infertile women referring for assisted reproduction. Nutrients.

[62] McGrath J (2001). Does ‘imprinting’ with low prenatal vitamin D contribute to the risk of various adult disorders?. Med Hypotheses.

[63] IOM (2011). Dietary reference intakes for calcium and vitamin D.

[64] Kalra P, Das V, Agarwal A, Kumar M, Ramesh V, Bhatia E, Gupta S, Singh S, Saxena P, Bhatia V (2012). Effect of vitamin D supplementation during pregnancy on neonatal mineral homeostasis and anthropometry of the newborn and infant. Br J Nutr.

[65] Brooke OG, Brown IR, Bone CD, Carter ND, Cleeve HJ, Maxwell JD, Robinson VP, Winder SM (1980). Vitamin D supplements in pregnant Asian women: effects on calcium status and fetal growth. Br Med J.

[66] Marya RK, Rathee S, Dua V, Sangwan K (1988). Effect of vitamin D supplementation during pregnancy on foetal growth. Indian J Med Res.

[67] Cockburn F, Belton NR, Purvis RJ, Giles MM, Brown JK, Turner TL, Wilkinson EM, Forfar JO, Barrie WJ, McKay GS (1980). Maternal vitamin D intake and mineral metabolism in mothers and their newborn infants. Br Med J.

[68] Delvin EE, Salle BL, Glorieux FH, Adeleine P, David LS: Vitamin D supplementation during pregnancy: effect on neonatal calcium homeostasis. J Pediatr 1986, 109(2):328-334. 10.1016/s0022-3476(86)80396-1 3488384

[69] Hossain N, Kanani FH, Ramzan S, Kausar R (2014). Obstetric and neonatal outcomes of maternal vitamin D supplementation: results of an open-label, randomized controlled trial of antenatal vitamin D supplementation in Pakistani women. J Clin Endocrinol Metab.

[70] Morales E, Rodriguez A, Valvi D, Iñiguez C (2015). Deficit of vitamin D in pregnancy and growth and overweight in the offspring. J Obes Int.

[71] Mallet E, Gugi B, Brunelle P, Henocq A, Basuyau JP, Lemeur H (1986). Vitamin D supplementation in pregnancy: a controlled trial of two methods. Obstet Gynecol.

[72] Yu CK, Sykes L, Sethi M, Teoh TG, Robinson S (2009). Vitamin D deficiency and supplementation during pregnancy. Clin Endocrinol.

[73] Roth DE, Perumal N, Al Mahmud A, Baqui AH (2013). Maternal vitamin D3 supplementation during the third trimester of pregnancy: effects on infant growth in a longitudinal follow-up study in Bangladesh. J Pediatr.

[74] Grant CC, Stewart AW, Scragg R, Milne T, Rowden J, Ekeroma A, Wall C, Mitchell EA, Crengle S, Trenholme A, Crane J, Camargo CA. Vitamin D During Pregnancy and Infancy and Infant Serum 25-Hydroxyvitamin D Concentration. Pediatrics. 2014;133(1)e143–e153. doi:10.1542/peds.2013-2602. 10.1542/peds.2013-2602 24344104

[75] Barrett H, McElduff A (2010). Vitamin D and pregnancy: an old problem revisited. Best Pract Res Clin Endocrinol Metab.

[76] Litonjua AA, Carey VJ, Laranjo N, Harshfield BJ, McElrath TF, O'Connor GT, Sandel M, Iverson RE, Jr., Lee-Paritz A, Strunk RC et al: Effect of prenatal supplementation with vitamin D on asthma or recurrent wheezing in offspring by age 3 years: the VDAART randomized clinical trial. JAMA 2016, 315(4):362-370. doi: 310.1001/jama.2015.18589. 10.1001/jama.2015.18589 PMC7479967 26813209

[77] Dawodu A, Davidson B, Woo JG, Peng YM, Ruiz-Palacios GM, de Lourdes GM, Morrow AL (2015). Sun exposure and vitamin D supplementation in relation to vitamin D status of breastfeeding mothers and infants in the global exploration of human milk study. Nutrients.

[78] Greer FR (2001). Fat-soluble vitamin supplements for enterally fed preterm infants. Neonatal Netw.

[79] Blencowe H, Cousens S, Oestergaard MZ, Chou D, Moller AB, Narwal R, Adler A, Vera Garcia C, Rohde S, Say L (2012). National, regional, and worldwide estimates of preterm birth rates in the year 2010 with time trends since 1990 for selected countries: a systematic analysis and implications. Lancet.

[80] Field DJ, Dorling JS, Manktelow BN, Draper ES (2008). Survival of extremely premature babies in a geographically defined population: prospective cohort study of 1994-9 compared with 2000-5. BMJ.

[81] Hollis BW, Roos BA, Draper HH (1981). Vitamin D and its metabolites in human and bovine milk. J Nutr.

[82] Soliman A, Salama H, Alomar S, Shatla E, Ellithy K, Bedair E (2013). Clinical, biochemical, and radiological manifestations of vitamin D deficiency in newborns presented with hypocalcemia. Indian J En- docrinol Metab.

[83] Gross ML, Tenenbein M, Sellers EA (2013). Severe vitamin D deficiency in 6 Canadian first nation formula-fed infants. Int J Cicumpolar Health.

[84] Hollis BW, Wagner CL (2006). Vitamin D deficiency during pregnancy: an ongoing epidemic. Am J Clin Nutr.

[85] Lee JM, Smith JR, Philipp BL, Chen TC, Mathieu J, Holick MF (2007). Vitamin D deficiency in a healthy group of mothers and newborn infants. Clin Pediatr (Phila).

[86] Nicolaidou P, Hatzistamatiou Z, Papadopoulou A, Kaleyias J, Floropoulou E, Lagona E, Tsagris V, Costalos C, Antsaklis A (2006). Low vitamin D status in mother-newborn pairs in Greece. Calcif Tissue Int.

[87] Mithal A, Wahl DA, Bonjour JP, Burckhardt P, Dawson-Hughes B, Eisman JA, El-Hajj Fuleihan G, Josse RG, Lips P, Morales-Torres J (2009). Global vitamin D status and determinants of hypovitaminosis D. Osteoporos Int.

[88] Hilger J, Friedel A, Herr R, Rausch T, Roos F, Wahl DA, Pierroz DD, Weber P, Hoffmann K (2014). A systematic review of vitamin D status in populations worldwide. Br J Nutr.

[89] Wahl DA, Cooper C, Ebeling PR, Eggersdorfer M, Hilger J, Hoffmann K, Josse R, Kanis JA, Mithal A, Pierroz DD (2012). A global representation of vitamin D status in healthy populations. Arch Osteoporos.

[90] Hogler W, Munns CF (2016). Rickets and osteomalacia: a call for action to protect immigrants and ethnic risk groups. Lancet Glob Health.

[91] Thacher TD, Pludowski P, Shaw NJ, Mughal MZ, Munns CF, Högler W. Nutritional rickets in immigrant and refugee children. Public Health Reviews. 2016;37(1):3. 10.1186/s40985-016-0018-3 PMC5810111 29450045

[92] Benitez-Aguirre PZ, Wood NJ, Biesheuvel C, Moreira C, Munns CF (2009). The natural history of vitamin D deficiency in African refugees living in Sydney. Med J Aust.

[93] Misra M. Vitamin D insufficiency and deficiency in children and adolescents. In: Uptodate, Post TW (Ed). Waltham: UpToDate; 2017.

[94] Harel Z, Flanagan P, Forcier M, Harel D (2011). Low vitamin D status among obese adolescents: prevalence and response to treatment. J Adolesc Health.

[95] Pazianas M, Butcher GP, Subhani JM, Finch PJ, Ang L, Collins C, Heaney RP, Zaidi M, Maxwell JD (2005). Calcium absorption and bone mineral density in celiacs after long term treatment with gluten-free diet and adequate calcium intake. Osteoporos Int.

[96] Atapattu N, Shaw N, Hogler W (2013). Relationship between serum 25-hydroxyvitamin D and parathyroid hormone in the search for a biochemical definition of vitamin D deficiency in children. Pediatr Res.

[97] Kroll M, Bi C, Garber C, Kaufman H, Liu D, Caston-Balderrama A, Clarke N, Xie M, Reitz R, Suffin S (2015). Temporal relationship between vitamin D status and parathyroid hormone in the United States. PLoS One.

[98] Holick M (2017). The vitamin D deficiency pandemic: approaches for diagnosis, treatment and prevention. Rev Endocr Metab Disord.

[99] Ross AC, Manson JE, Abrams SA, Aloia JF, Brannon PM, Clinton SK, Durazo-Arvizu RA, Gallagher JC, Gallo RL, Jones G (2011). The 2011 report on dietary reference intakes for calcium and vitamin D from the Institute of Medicine: what clinicians need to know. J Clin Endocrinol Metab.

[100] Committe, Opinion, #495 (2011). Vitamin D: screening and supplementation during pregnancy. Obstet Gynecol.

[101] Nice Guideline: Vitamin D: increasing supplement use in at-risk group. In., vol. PH56. United Kingdom: National Institute for Health and Care Excellence; 2014.

[102] Braegger C, Campoy C, Colomb V, Decsi T, Domellof M, Fewtrell M, Hojsak I, Mihatsch W, Molgaard C, Shamir R (2013). Vitamin D in the healthy European paediatric population. J Pediatr Gastroenterol Nutr.

[103] Specker BL, Ho ML, Oestreich A, Yin TA, Shui QM, Chen XC, Tsang RC (1992). Prospective study of vitamin D supplementation and rickets in China. J Pediatr.

[104] Beser E, Cakmakci T (1994). Factors affecting the morbidity of vitamin D deficiency rickets and primary protection. East Afr Med J.

[105] Misra M, Pacaud D, Petryk A, Collett-Solberg PF, Kappy M (2008). Vitamin D deficiency in children and its management: review of current knowledge and recommendations. Pediatrics.

[106] Canadian Paediatric Society. Vitamin D supplementation: recommendations for Canadian mothers and infants. Paediatr Child Health. 2007;12(7):583–98. PMC2528771 19030432

[107] Calvo MS, Whiting SJ, Barton CN (2005). Vitamin D intake: a global perspective of current status. J Nutr.

[108] Erkkola M, Nwaru BI, Viljakainen HT (2011). Maternal vitamin D during pregnancy and its relation to immune-mediated diseases in the offspring. Vitam Horm.

[109] Uday S, Kongjonaj A, Tulchinsky T, Hogler W Variations in infant and childhood Vitamin D supplementation programs across Europe and factors influencing adherence. Endocrine Abstracts. 2016;45:OC5.4. 10.1530/EC-17-0193 PMC5655685 28924002

[110] Williamson S, Greene S (2007). Rickets: prevention message is not getting through. BMJ.

[111] Ahmed SF, Franey C, McDevitt H, Somerville L, Butler S, Galloway P, Reynolds L, Shaikh MG, Wallace AM (2011). Recent trends and clinical features of childhood vitamin D deficiency presenting to a children's hospital in Glasgow. Arch Dis Child.

[112] Taylor JA, Geyer LJ, Feldman KW (2010). Use of supplemental vitamin d among infants breastfed for prolonged periods. Pediatrics.

[113] Perrine CG, Sharma AJ, Jefferds ME, Serdula MK, Scanlon KS (2010). Adherence to vitamin D recommendations among US infants. Pediatrics.

[114] Lande B, Andersen LF, Baerug A, Trygg KU, Lund-Larsen K, Veierod MB, Bjorneboe GE (2003). Infant feeding practices and associated factors in the first six months of life: the Norwegian infant nutrition survey. Acta Paediatr.

[115] Dratva J, Merten S, Ackermann-Liebrich U (2006). Vitamin D supplementation in Swiss infants. Swiss Med Wkly.

[116] Hollis BW, Wagner CL, Howard CR, Ebeling M, Shary JR, Smith PG, Taylor SN, Morella K, Lawrence RA, Hulsey TC: Maternal versus infant vitamin D supplementation during lactation: a randomized controlled trial. Pediatrics 2015, 136(4):625-634. doi: 610.1542/peds.2015-1669. 10.1542/peds.2015-1669 PMC4586731 26416936

[117] Oberhelman SS, Meekins ME, Fischer PR, Lee BR, Singh RJ, Cha SS, Gardner BM, Pettifor JM, Croghan IT, Thacher TD: Maternal vitamin D supplementation to improve the vitamin D status of breast-fed infants: a randomized controlled trial. Mayo Clin Proc 2013, 88(12):1378-1387. doi: 1310.1016/j.mayocp.2013.1309.1012. 10.1016/j.mayocp.2013.09.012 PMC3923377 24290111

[118] Cooper C, Harvey NC, Bishop NJ, Kennedy S, Papageorghiou AT, Schoenmakers I, Fraser R, Gandhi SV, Carr A, D'Angelo S (2016). Maternal gestational vitamin D supplementation and offspring bone health (MAVIDOS): a multicentre, double-blind, randomised placebo-controlled trial. Lancet Diabetes Endocrinol.

[119] Hollis BW, Johnson D, Hulsey TC, Ebeling M, Wagner CL (2011). Vitamin D supplementation during pregnancy: double-blind, randomized clinical trial of safety and effectiveness. J Bone Miner Res.

[120] Dawodu A, Saadi HF, Bekdache G, Javed Y, Altaye M, Hollis BW (2013). Randomized controlled trial (RCT) of vitamin D supplementation in pregnancy in a population with endemic vitamin D deficiency. J Clin Endocrinol Metab.

[121] Zipitis CS, Markides GA, Swann IL (2006). Vitamin D deficiency: prevention or treatment?. Arch Dis Child.

[122] Du X, Zhu K, Trube A, Zhang Q, Ma G, Hu X, Fraser DR, Greenfield H (2004). School-milk intervention trial enhances growth and bone mineral accretion in Chinese girls aged 10-12 years in Beijing. Br J Nutr.

[123] Serra-Majem L (2001). Vitamin and mineral intakes in European children. Is food fortification needed?. Public Health Nutr.

[124] Nowson CA, Margerison C (2002). Vitamin D intake and vitamin D status of Australians. Med J Aust.

[125] Hayes A, Cashman KD (2017). Food-based solutions for vitamin D deficiency: putting policy into practice and the key role for research. Nutr Soc.

[126] Moy RJ, McGee E, Debelle GD, Mather I, Shaw NJ (2012). Successful public health action to reduce the incidence of symptomatic vitamin D deficiency. Arch Dis Child.

[127] Grant WB (2011). An estimate of the global reduction in mortality rates through doubling vitamin D levels. Eur J Clin Nutr.

[128] Palacios C, Gonzalez L. Is vitamin D deficiency a major global public health problem? The Journal of Steroid Biochemistry and Molecular Biology. 2014;144PA:138–45. doi:10.1016/j.jsbmb.2013.11.003. 10.1016/j.jsbmb.2013.11.003 PMC4018438 24239505

[129] Wagner CL, McNeil RB, Johnson DD, Hulsey TC, Ebeling M, Robinson C, Hamilton SA, Hollis BW (2013). Health characteristics and outcomes of two randomized vitamin D supplementation trials during pregnancy: a combined analysis. J Steroid Biochem Mol Biol.

